# Cost-Optimization-Based Quantum Key Distribution over Quantum Key Pool Optical Networks

**DOI:** 10.3390/e25040661

**Published:** 2023-04-14

**Authors:** Jie Jia, Bowen Dong, Le Kang, Huanwen Xie, Banghong Guo

**Affiliations:** 1 Guangdong Provincial Key Laboratory of Nanophotonic Functional Materials and Devices, Guangdong Provincial Key Laboratory of Quantum Engineering and Quantum Materials, South China Normal University, Guangzhou 510006, China; 2 National Quantum Communication (Guangdong) Co., Ltd., Guangzhou 510700, China

**Keywords:** trusted and untrusted relay, measurement-device-independent quantum key distribution, optical networks, quantum key pool, cost optimization

## Abstract

The Measurement-Device-Independent-Quantum Key Distribution (MDI-QKD) has the advantage of extending the secure transmission distances. The MDI-QKD combined with the Hybrid-Trusted and Untrusted Relay (HTUR) is used to deploy large-scale QKD networks, which effectively saves deployment cost. We propose an improved scheme for the QKD network architecture and cost analysis, which simplifies the number of QKD transmitters and incorporates the quantum key pool (QKP) in the QKD network. We developed a novel Hybrid-QKD-Network-Cost (HQNC) heuristic algorithm to solve the cost optimization problem. Simulations verified that the scheme in this paper could save the cost by over 50 percent and 90 percent, respectively.

## 1. Introduction

Quantum Key Distribution (QKD) [1,2] technology has caused abundant research and attention from academia and industry. The expansion of multi-user networks [3,4] is an inevitable trend. The transmission distance is from the initial few meters to today’s hundreds [5] and even thousands of kilometers [6], as well as the secret key rate from the bit to the Mbps [7] level.

There are many more technologies to widely deploy QKD networks: quantum relay, trusted relay, untrusted relay, and optical switch. In recent years, quantum relay [8,9,10] technology has made significant progress, but its development has been mainly concentrated on the laboratory setting [9], which is still some distance away from real practicality. The trusted relay [11,12] is the most-widely used relaying technology and the most-mature one. The untrusted relay [13] provides higher security, but limited transmission distance. QKD networks based on optical switches [14] are easy to implement. The optical switch is used as an intermediate node to extend the QKD secure transmission distance within a certain distance range and reduce the deployment cost of QKD networks. Yet, the application range is small and limited by the distance, as well as the number of users.

Multiple-user [3,15] QKD networks are crucial for realizing secure communication in a practical environment. The United States, Europe, and Japan are actively laying out quantum communication networks and applying the to various fields. In fact, the above QKD networks are based on the trusted relay and optical switch. The deployment of quantum relays has not yet been implemented in a mature network. A detailed comparison is given in Table 1.

The Measurement-Device-Independent-Quantum Key Distribution (MDI-QKD) [19,20] closes all detector vulnerabilities and doubles the transmission distance. Based on existing networks, the MDI-QKD [13] network with the Hybrid Trusted and Untrusted Relay (HTUR) extends the transmission distance and improves security further, where untrusted relays do not rely on any assumptions on measurement. We note the introduction of the Quantum Key Pool (QKP) [4,21,22,23] in QKD networks. The secret keys generated between QKD node pairs are stored in the QKP temporarily to reduce the waste of secret keys. The QKP increases the secret key resource utilization, which thoroughly lowers the deployment of crucial QKD devices and saves costs.

Hence, how to efficiently deploy HTUR QKD networks with the QKP is an essential problem. In this paper, we addressed this issue and focused on the optimization of the deployment cost with the Hybrid QKD Network cost (HQCN) heuristic algorithm. Our main contributions are as follows

(I) We introduced a new four-layer architecture of the QKD network, in which the QKPs are deployed in the QKD layer temporarily to store the secret keys generated between QKD node pairs.

(II) We propose a new HTUR QKD network structure with the QKP used to illustrate the long-distance secret keys’ transmission.

(III) We established a new HTUR QKD network cost model where the deployment costs of various QKD devices are considered.

(IV) We designed an HQNC heuristic algorithm to evaluate the performance through numerical simulation and performed a comparative analysis with the Cost-Optimized QKD Backbone Networking (CO-QBN) and Purely Trusted Relay (PTR) schemes in terms of network device depletion and total deployment cost, respectively.

The rest of this paper is structured as follows: Section 2 briefly describes the QKD network architecture and node structure. Section 3 defines the network model and cost model. Section 4 proposes an efficient HQNC heuristic algorithm, and performs extensive numerical simulations for the performance evaluation. Finally, Section 5 gives the conclusion.

## 2. QKD Network Architecture

**Figure 1:**
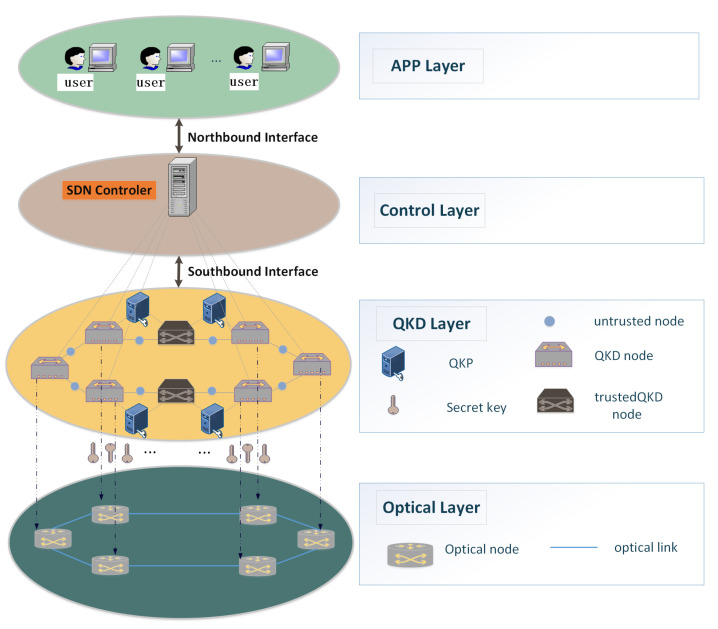
QKD network architecture.

In this section, we introduce a QKD network architecture, as shown in Figure 1. This network architecture consists of a four-layer structure: the Application layer (APP layer), the control layer, the QKD layer, and the optical layer, respectively. Compared with Cao et al. [24], the architecture in this paper adopts the MDI-QKD [19,25,26] protocol and introduces the HTUR for cooperative deployment. The QKPs tentatively store the secret keys generated by the two neighboring nodes. The abbreviations and definitions in this paper are listed in Table 2.

The four layers of the structure collaborate with each other to complete the unconditional secure information encryption transmission. In the APP layer, multiple QKD users randomly send out secret key requests, and each user with specific security requirements requests a certain number of secret keys from the QKD layer. The Software-Defined Network (SDN) controller receives the QKD user’s requests through the northbound interface [27] (restful Application Programming Interface (API) protocol). The user requests with a high secret key demand are prioritized and sent to the QKD layer and optical layer via the southbound interface [28] (open flow protocol). In the QKD layer, three different nodes are required: the QKD nodes, the trusted relay nodes, and the untrusted relay nodes. Secret keys are generated between neighboring QKD nodes and trusted relay nodes or between two neighboring trusted relay nodes. Untrusted relay nodes are third-party nodes. The QKD layer sends the secret keys located in the QKP to the optical layer, which is co-located with the QKD layer. Then, the encryption of the security information requested by a specific user is achieved. Finally, the SDN controller sends the completion command to the application layer.

**Figure 2:**
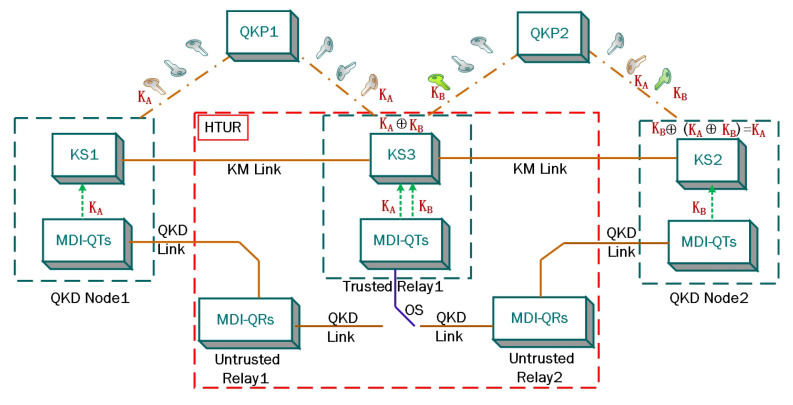
Structure of HTUR QKD layer based on the QKP.

As shown in Figure 2, the trusted relay nodes include the Measurement-Device-Independent-Quantum Transmitters (MDI-QTs), the Key Service (KS), and the Optical Switches (OSs). When the OS and Untrusted Relay 1 are connected, a pair of secret keys $K_{A}$ is generated between QKD Node 1 and Trusted Relay Node 1, and the generated key pair $K_{A}$ is temporarily stored in QKP1. When the OS and Untrusted Relay 2 are connected, a pair of secret keys $K_{B}$ is generated between QKD Node 2 and Trusted Relay Node 1, and the generated secret key pair $K_{B}$ is temporarily stored in QKP2. For the secret key requirements of more distant node pairs, the staggered deployment of the HTUR is added to the QKD links. Note that only one MDI-QTs device is contained in Trusted Relay Node 1, and we would flexibly utilize the MDI-QTs located in Trusted Relay Node 1 by controlling the OS. The secret keys’ transfer process between the remote QKD Node 1 and QKD Node 2 is as follows: the $K_{A}$ and $K_{B}$ located in KS3 conduct the bitwise exclusive OR operation, and $k_{A}$ ⨁ $K_{B}$ is sent to KS2 through a key management (KM) link. According to $K_{B}$ ⨁ ($K_{A}$ ⨁ $K_{B}$) = $K_{A}$, the KS2 retrieves $K_{A}$. Then, both KS1 and KS2 would access $K_{A}$. Hence, $K_{A}$ is shared between QKD Node 1 and QKD Node 2.

In the QKD network, the Public Channel (PCh) and the Data Channel (DCh) deploy Erbium-Doped Fiber Amplifiers (EDFAs) at every distance of 80 km (m = 80 km) [29] on the optical link to enable the long-distance transmission of optical signals, where m denotes the distance between the adjacent QKD node/trusted relay and the untrusted relay. In this paper, we reference the previous literature [13,30]: when the untrusted relay or the trusted relay is deployed at the same physical location as the EDFA, only one Multiplex/Demultiplex (MOD) component can be used for both the EDFA bypass [31] and the multiplexing/demultiplexing QKD, KM, and optical links. Hence, to save the MOD component, we assumed that the trusted and untrusted relays are deployed at the same location as the EDFAs. In the MDI-QKD network, the distances of two adjacent MDI-QTs and trusted–untrusted relays are 160 km and 80 km, respectively.

## 3. Problem and Algorithm Formulation

In this section, we formulate the cost model and the algorithm model to elaborate the QKD network cost less problem. The notations used in this paper are listed and defined in Table 3.

### 3.1. QKD Network Model

For ensure the isolation, MODs are utilized in different QKD and KM links in the same fiber [32]. We assumed that the QKD nodes have a co-located deployment with optical nodes. Hence, the topology of the QKD layer is similar to the optical layer. We modeled the network topology as G(N,L), where N and L denote the set of optical/QKD nodes and fiber links, respectively. The QKD links consist of Quantum Channels (QChs) and PChs, and the KM links contain only classical channels. The fiber links in the optical layer comprise the DChs. Different wavelength channels are allocated in the QKD links, where the sets of wavelengths for the quantum and classical channels are denoted as $W_{Q}$ and $W_{KM}$, respectively.

### 3.2. Cost Model

To better illustrate the cost optimization of the QKD network, before analyzing the cost of the QKD network, we define a QKD request parameter $r(s_{r},d_{r},P_{r})$, where $s_{r}$ and $d_{r}$ denote the source and destination nodes of the QKD request r, respectively, and $P_{r}$ denotes the number of parallel QKD links that satisfy the secret key rate requirement of the QKD requests. According to the principle of wavelength division multiplexing [32], multiple parallel QKD links could be multiplexed in the same fiber to achieve a higher secret key rate and save fiber resources. $P_{r}$ is defined as

(1) $$P_{r}=E_{r}/E_{D}$$

where $E_{r}$ denotes the secret key rate requirement for a QKD request r. $E_{D}$ denotes the secret key rate at distance D (D = 2 m) [13], where D represents the distance of a pair of adjacent MDI-QTs. The untrusted relay is placed in a symmetric position of a pair of MDI-QTs. The specific secret key rate of each QKD link was not considered in this work, and here, we only conducted a quantitative analysis.

Based on the network topology, we define a parameter:

(2) $$\eta =\frac{N(N-1)}{2}$$

Here, η denotes the total number of QKD link requests in the network when any QKD node pair hosts one request. N denotes the number of optical/QKD nodes.

The costs of QKD network deployment come from the various devices in the network that support the HTUR with QKP over the network. This section details the following:

(1) Number of MDI-QTs and MDI-QRs and cost:

In this paper, we utilized only the MDI-QKD transceiver to implement the QKD for the whole network. Two MDI-QTs and one MDI-QRs are required to complete a QKD process, due to the MDI-QKD protocol being adopted in the QKD optical network. Therefore, the number of MDI-QTs and MDI-QRs required for an MDI-QKD request is expressed as

(3) $$M_{QT}^{r}=\left(\left\lceil \frac{L_{sd}}{D}\right\rceil +1\right)\cdot P_{r}$$

(4) $$M_{QR}^{r}=\left\lceil \frac{L_{sd}}{D}\right\rceil \cdot P_{r}$$

where the Gaussian brackets here indicate upward rounding, $L_{sd}$ is the physical length of the fiber links between the source node s and the destination node d of the adjacent QKD nodes, $P_{r}$ is the number of parallel QKD links of r, and D is the distance between a pair of connected MDI-QTs. Based on (3) and (4), the cost of the MDI-QTs and MDI-QRs for all QKD requests R can be expressed as

(5) $$C_{QT}^{R}=\sum_{r\epsilon R}M_{QT}^{r}\cdot \delta_{QT}^{r}$$

(6) $$C_{QR}^{R}=\sum_{r\epsilon R}M_{QR}^{r}\cdot \delta_{QR}^{r}$$

where $\delta_{QT}^{r}$ and $\delta_{QR}^{r}$ denote the cost of an MDI-QTs and MDI-QRs for QKD request r, respectively.

(2) Number of KSs and cost:

From Figure 2, the Key Services (KSs) are placed at the same physical location as the QKD transmitters where the number of KSs required for a QKD request is equal to the MDI-QKD transmitter. Thus, the number of parallel QKD links is not considered when calculating the number of KSs. The number of KSs required for a QKD request r can be expressed as

(7) $$M_{KS}^{r}=\left(\left\lceil \frac{L_{sd}}{D}\right\rceil +1\right)$$

In order to ensure the security of the secret key storage, we assumed that the KS is deployed independently for each QKD request. Therefore, the KS cost of all QKD links in the network can be expressed as

(8) $$C_{KS}^{R}=\sum_{r\epsilon R}M_{KS}^{r}\cdot \delta_{KS}^{r}$$

(3) Number and cost of QKPs:

The QKP [21] technique can achieve the effective management of precious secret key resources and improve the efficiency of the quantum key distribution. Reference [24] showed that the QKPs are abstraction rather than independent devices. Therefore, the specific cost of the QKP was not considered here.

(4) Number and cost of MODs:

To save fiber deployment and reduce the waste of fiber resources, we added MODs to coexist with the QKD links and KS links as single fibers. Our specific implementation was to deploy MOD [20] devices at QKD nodes and trusted and untrusted relay nodes. The equation is expressed as follows:

(9) $$M_{MOD}^{r}=\left(2\cdot \left\lceil \frac{L_{sd}}{D}\right\rceil +1\right)$$

Then, the cost of the MODs required for all requests R can be expressed as

(10) $$C_{MOD}^{R}=\sum_{r\epsilon R}M_{MOD}^{r}\cdot \delta_{MOD}^{r}$$

(5) QKD and KM link costs:

In this section, we know there are two channels in the QKD link from Figure 2: the QChs and the PChs are used for quantum key generation and post-processing [31] between QKD nodes, respectively. Besides, there is the KM link along another wavelength channel for long-distance transmission secret keys. Then, the physical length of the QKD and KM links required for a QKD request r is expressed as

(11) $$L_{r}=\left(2\cdot P_{r}\cdot L_{sd}+L_{KM}\right)$$

Here, $P_{r}$ denotes the number of parallel QKD links, and $L_{sd}$ denotes the distance between source node s and destination node d of QKD request r. Parameter 2 refers to a quantum channel and a classical channel included in the QKD link. $L_{KM}$ indicates the physical length of the KM links. We note that the KM and the QKD links’ length are equal under the same requests.

Based on Equation (11), the cost of the QKD and KM link for all QKD requests R can be expressed as

(12) $$C_{L}^{R}=\sum_{r\epsilon R}L_{r}\cdot \delta_{L}^{r}$$

where $\delta_{L}^{r}$ denotes the QKD and KM links’ cost for each QKD request r.

(6) Total deployment cost:

The total cost of deploying the HTUR QKD network with the QKP is expressed as

(13) $$C_{R}=C_{QT}^{R}+C_{QR}^{R}+C_{L}^{R}+C_{MOD}^{R}+C_{KS}^{R}$$

The total cost values are calculated based on Equations (5)–(8), (10), (12), and (13), respectively. Other auxiliary equipment (e.g., OSs, etc.) was not considered in this study owing to its low cost value.

### 3.3. Heuristic Algorithm

Based on the above HTUR QKD network and cost models, we propose an HQNC heuristic algorithm. The details are given in Algorithm 1. We first initialized the parameters $C_{R}$, $C_{r}$, $M_{QT}^{r}$, $M_{QR}^{r}$, $M_{KS}^{r}$, and $L_{r}$. For each arriving QKD request r, the Dijkstra [21,31] algorithm was applied to select the shortest path Or for r, and the length Lsd of the shortest path was calculated after the shortest path was obtained. In the next step, the number of parallel QKD channel wavelengths required under each QKD request r was obtained from the equation $P_{r}$=$E_{r}/E_{D}$. After obtaining the shortest path and the required number of wavelengths for a QKD request, we applied the first-fit [21] algorithm to assign the appropriate wavelengths (QKD wavelengths and KM channel wavelengths) to the QKD request r. The first-fit algorithm has the advantage of low computational complexity, and it selects the wavelengths in the lower position among all accessible wavelength sets.

After both the QKD path and the available channel wavelength are defined, the number of MDI-QTs, MDI-QRs, MODs, and KSs and the length of the wavelength links under each QKD request are calculated according to Equations (3), (4), (7), (9), and (11), respectively. The corresponding parts of the heuristic algorithm in Algorithm 1 are Rows 14 to 21. The cost of MDI-QTs, MDI-QRs, MODs, and KSs and the length of the wavelength links for all QKD requests in the network can be calculated by Equations (5), (6), (8), (10), and (12), respectively. Further, the total deployment cost in the QKD network is obtained from Equation (13). The corresponding parts of the HQNC algorithm in Table 1 are Rows 23 and 30.
**Algorithm 1: Hybrid QKD Network Cost (HQNC) Algorithm.****Input: **$G\left(N,L\right)$, R, D, $W_{Q}$, $W_{KM}$, $\delta_{QT}^{r}$, $\delta_{QR}^{r}$, $\delta_{KS}^{r}$, $\delta_{L}^{r}$, $W_{MOD}$.**Output: **$C_{R}$, $C_{r}$ routing, and wavelength allocation for each QKD request, update QKD network’s state:1. Initialize $C_{R}\leftarrow 0$; $C_{r}\leftarrow 0$.2. **For** each QKD network request rϵR, do:3.    Initialize $M_{QT}^{r}\leftarrow 0$, $M_{QR}^{r}\leftarrow 0$, $M_{KS}^{r}\leftarrow 0$, $L_{r}\leftarrow 0$.4.    Routing computation with Dijkstra’s shortest path algorithm.5.    Compute the physical distance $L_{sd}$ between the QBN source node $S_{r}$ and destination node      $d_{r}$ of the r on the shortest path Or.6.    Compute the required number of QChs $P_{r}=E_{r}/E_{D}$.7.    Search all available wavelength channels, and store them in $W_{Q}$ as the QChs on the shortest      path Or.8.    **If** $\left|W_{Q}\right|\geq 2\cdot P_{r}$, **then**9.       Filter $2\cdot P_{r}$ wavelength channels from $W_{Q}$ using first-fit algorithm for QKD request r.10.    Search all available wavelength channels, and store them in $W_{KM}$ as the KM links on the         shortest path Or.11.   **If** $\left|W_{KM}\right|\geq 1$, **then**12.             filter one wavelength channel from $W_{KM}$ using first-fit algorithm for QKD request r.13.             **For** the shortest path Or of QKD request r, **do:**14.                  Compute the required number of QKD transmitters and R:15.                         $M_{QT}^{r}\leftarrow \left(\lceil L_{sd}/D\rceil +1\right)\cdot P_{r}$;16.                         $M_{QR}^{r}\leftarrow \left\lceil L_{sd}/D\right\rceil \cdot P_{r}$.17.                   Compute the required number of KSs and MODs of the QKD request r:18.                         $M_{KS}^{r}\leftarrow \left(\left\lceil L_{sd}/D\right\rceil \right)\cdot P_{r}$;19.                         $M_{MOD}^{r}\leftarrow \left(2\cdot \left\lceil L_{sd}/D\right\rceil \right)\cdot P_{r}$.20.                   Compute the required length of links of the QKD request located in:21.                              $L_{r}\leftarrow 2\cdot P_{r}\cdot L_{sd}+L_{KM}$.22.               **End for**23.               The total cost:24.                        $C_{R}\leftarrow C_{r}+M_{QT}^{r}\cdot \delta_{QT}^{r}+M_{QR}^{r}\cdot \delta_{QR}^{r}+M_{MOD}^{r}\cdot \delta_{MOD}^{r}+M_{KS}^{r}\cdot \delta_{KS}^{r}+L_{r}\cdot \delta_{L}^{r}$.25.       **Else**:26.         The secret-key rate demand of QKD request r cannot be satisfied.27.   **Else**:28.      The secret-key rate demand of QKD request r cannot be satisfied.29. **End**30. The total cost for all QKD requests $C_{R}\leftarrow C_{R}+C_{r}$.31. **Return** $C_{R}$, $C_{r}$ routing, and wavelength allocation for each QKD request, and update QKD links’ state.

In the implementation process in detail, the time complexity of this algorithm mainly depends on the size of the network and the constraints. In the worst case, the time complexity of Rows 3–9 and Rows 10–36 in this algorithm is $O(|N{|}^{2})$ and $O(W_{Q}\cdot W_{KM})$, respectively. Therefore, the total time complexity of this HQNC algorithm is $O(|N{|}^{2}+W_{Q}\cdot W_{KM})$.

## 4. Performance Evaluation and Analysis

**Figure 3:**
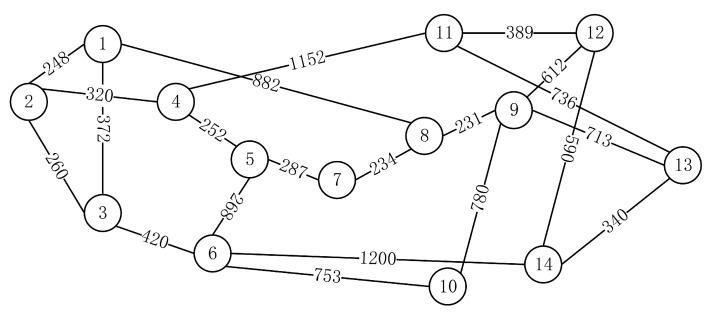
NSFNET topology.

**Figure 4:**
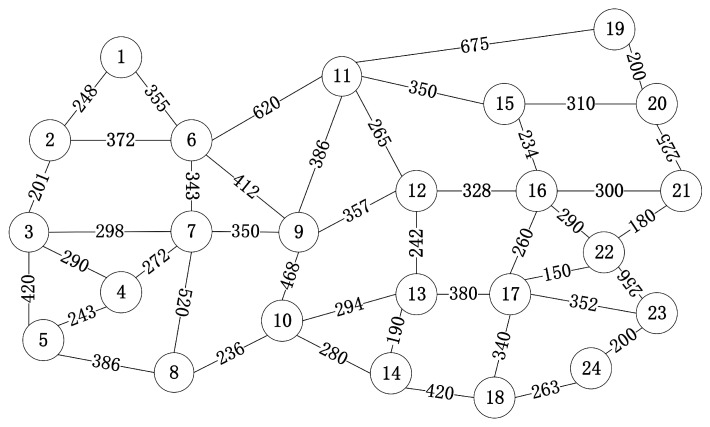
USNET topology.

In this section, we introduce the HTUR QKD network with the QKP for cost optimization. To illustrate the efficiency and feasibility of the scheme, we conducted an extensive simulation analysis, with PTR [31] and CO-QBN [13] for comparison. Further, the National Science Foundation Network (NSFNET) and United States Network (USNET) topologies shown in Figure 3 and Figure 4 were adopted for the deployment network cost. QKD requests were randomly generated among all QKD node pairs, and the maximum number of QKD requests satisfied by various QKD networks could be calculated according to the formula |N|·(|N|−1)/2. Thus, the maximum number of QKD requests on the NSFNET and USNET is 91 and 276, respectively. We considered the case that there will be more QKD requests in the near future and the existing network users’ scale may need to be further expanded. For the simulation, we assumed that the range of QKD requests on the NSFNET and USNET topologies was [20, 200] and [40, 400], respectively. The weight values of two neighboring nodes on the network topology refer to the physical distance (kilometers). We assumed that there are sufficient wavelengths in the links and there is no case of an insufficient number of wavelengths and request allocation failure. To satisfy the different secret key requirements for the QKD requests, the secret key rate was set as {k}, {k,2k}, and {k,2k,3k} respectively.

The cost values used for the performance evaluation are listed in Table 4. These cost values required in the QKD network were based on reasonable assumptions, which were provided by the vendor. Considering the price fluctuations in cost values due to component reduction as technology advances, we propose two scenarios to analyze the performance: (1) Current Scenario (CS): The cost value of each component is fixed. This is applicable to the current mature deployment of the network. (2) Future Scenario (FS): The cost value of each component is also fixed, but the cost value of the FS is extremely reduced compared to the CS. This scenario helped us to analyze the long-term impact of device cost optimization in the network. The problem of predicting future network costs can be better addressed.

### 4.1. QKD Network Critical Device Requirements Analysis

In this section, we evaluated in detail the various device deployment issues for QKD networks and adopted the number of required QKD transmitters and receivers as examples. Furthermore, we utilized TRN and CO-QBN as the comparison schemes to specify the efficiency of our new proposed HQCN scheme.

#### QKD Transmitter and Receiver Number

**Figure 5:**
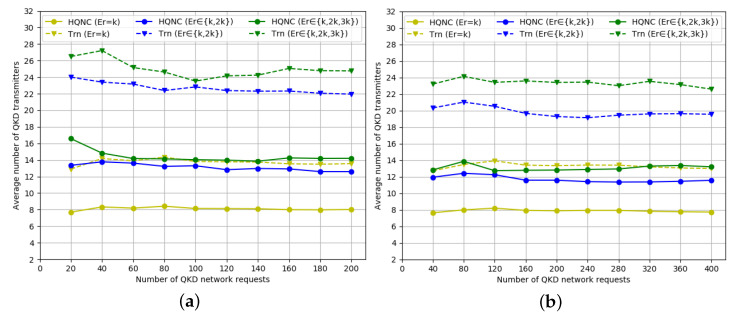
Average number of QKD transceivers versus number of QKD requests compared to TRN based on (**a**) NSFNET topology and (**b**) USNET topology.

**Figure 6:**
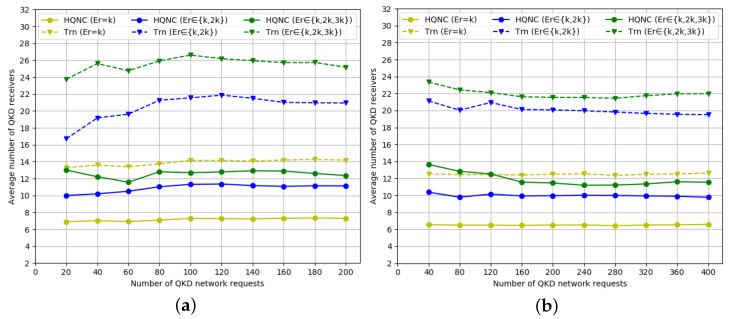
Average number of QKD receivers versus number of QKD requests compared with TRN based on (a) NSFNET topology and (b) USNET topology.

Figure 5 and Figure 6 demonstrate the results between the average number of QKD transmitters/receivers (per QKD request) and QKD network requests under the NSFNET and USNET topologies, respectively. The PTR scheme was used for comparison in Figure 5 and Figure 6. As the secret key rate of QKD requests increases, the average number of corresponding QKD transmitters also increases, and the trend of increasing the number of QKD transmitters and receivers based on both topologies is consistent. The result is that, according to Equations (3) and (4), the average number of QKD transmitters and receivers is related to the secret key rate requirement.

The average number of QKD transmitters required for QKD requests do not show a corresponding linear increase as the QKD requests increase when the secret key rate is {k}, {k,2k}, and {k,2k,3k}, respectively, that is QKD requests are randomly generated among all QKD node pairs. As shown in Figure 5a and Figure 6a, the average number of QKD transmitters/receivers required by the HQNC is smaller than the PTR scheme, which also proves the efficiency of our new proposed method. The advantage of a reduced transmitters/receivers number is especially obvious when the secret key rate is higher. The reason is that the PTR scheme requires more QKD transmitting/receiving devices compared to the HQNC scheme when the secret key rate increases. The number of transmitters/receivers required under the same QKD requests number is slightly larger than the number of receivers. The result is that, adopting the HTUR QKD with the QKP incorporating the MDI-QKD protocol, one more QKD transmitter than QKD receivers is required to complete a QKD request.

From Figure 5b and Figure 6b, we observe that the number of QKD transmitters/receivers shows an irregular variation as the network scale increases. The main reasons are that the average calculation method is used to compute the number of QKD transmitters and receivers, the QKD requests are generated randomly, and the distances between different QKD node pairs are different, and none of the above reasons is related to the network scale.

Therefore, the HQNC algorithm can reduce the number of QKD transmitters and receivers to deploy QKD networks to a larger extent; especially when the secret key rate is higher, the advantages of the HQNC scheme are more obvious. Moreover, the HQNC algorithm for reducing QKD transmitters and receivers is related to the distance between QKD node pairs in the network rather than to the network topology scale.

**Figure 7:**
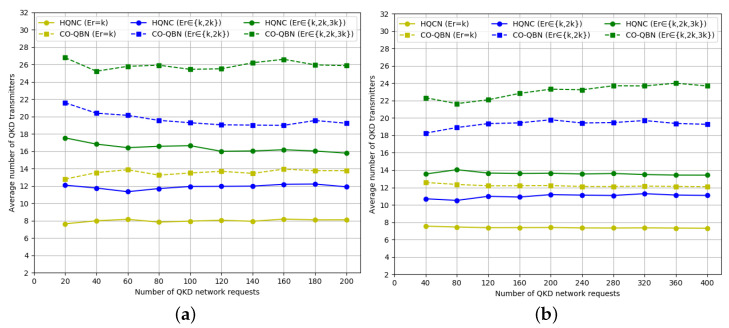
Average QKD transceivers number versus QKD requests number compared to CO-QBN based on (**a**) NSFNET topology and (**b**) USNET.

**Figure 8:**
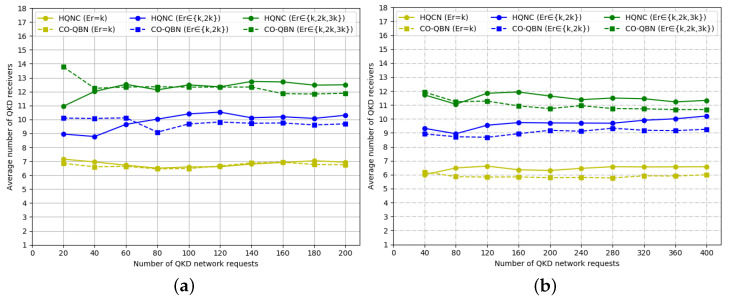
Average QKD receivers number versus QKD requests number compared to CO-QBN based on (**a**) NSFNET topology and (**b**) USNET.

From Figure 7a,b, the number of QKD transmitters in the HQNC is significantly reduced in both the NSFNET and USNET topologies compared to CO-QBN. According to Figure 8a,b, the number of QKD receivers for the HQNC cost optimization solution is similar to the CO-QBN algorithm. The result is that we mainly reduced the deployment of QKD transmitters compared to the CO-QBN algorithm in the network.

### 4.2. QKD Network Total Deployment Cost Analysis

In this section, we analyzed the QKD network total deployment cost problem in detail. To reflect the generality of the deployment cost, we considered two scenarios (CS and FS) separately, as described below

**Figure 9:**
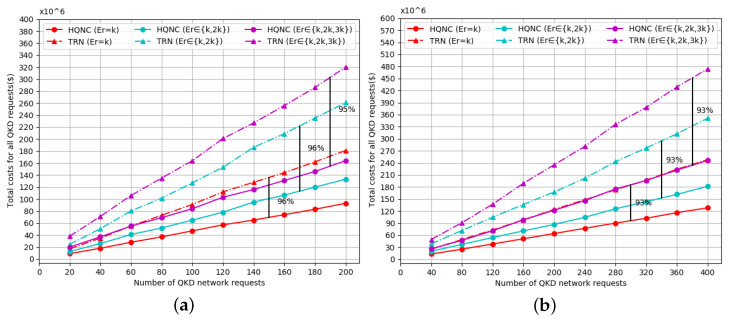
Total QKD network cost versus number of QKD requests compared to TRN in the current scenario based on (**a**) NSFNET topology and (**b**) USNET topology.

**Figure 10:**
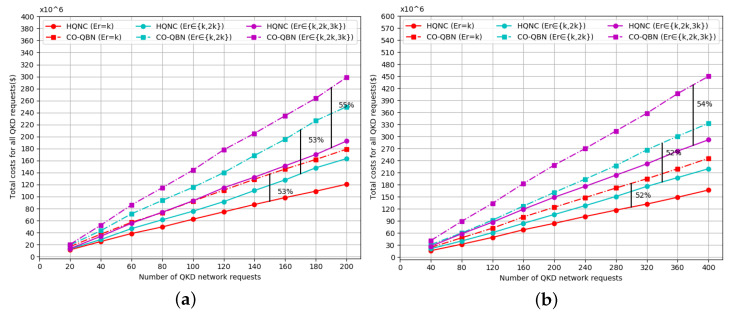
Total QKD network cost versus number of QKD requests compared to CO-QBN in the current scenario based on (**a**) NSFNET topology and (**b**) USNET topology.

**Figure 11:**
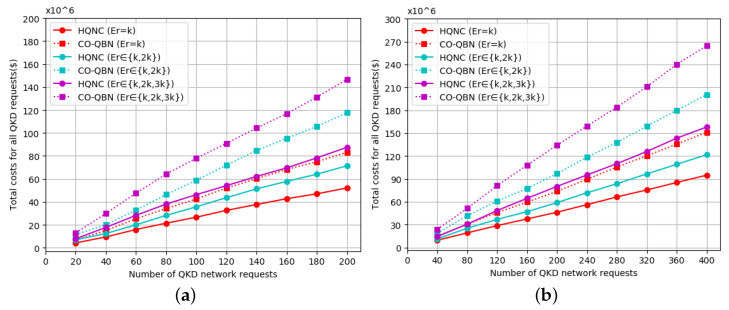
Total QKD network cost versus number of QKD requests compared to CO-QBN in the future scenario based on (**a**) NSFNET topology and (**b**) USNET topology.

The total deployment costs of QKD network requests are shown in Figure 9, Figure 10 and Figure 11, respectively. Figure 9 and Figure 10 show the total QKD request deployment cost on the NSFNET and USNET topologies for the CS, with TRN and CO-QBN as comparisons, respectively. Figure 11 shows the total QKD requests’ deployment cost on the NSFNET and USNET network topologies for the FS, compared with the HTUR only.

According to Figure 9 and Figure 10, it can be observed that the total cost value gradually increases as the number of QKD requests increases. In the same scenario (as shown in Table 2), the total cost value improves gradually with the increase of the secret key rate. Compared with the previous TRN and CO-QBN schemes, the HQNC in this paper requires a significantly lower total cost value for the same secret key rate. This phenomenon verifies the efficiency of our new proposed scheme. In Figure 9a,b, the TRN curve is always higher than that of the HQNC when the secret key rate is fixed. As the number of QKD requests increases, the difference of the cost values between TRN and HQNC becomes larger. The reason is that, as the number of QKD requests increases, the cost savings become greater in HQNC compared to TRN. The total cost of QKD network deployment increases gradually with the expansion of the network scale, which is due to the number of QKD requests accommodated. In the CS, the cost savings of the presented HQNC are more than 90 percent and 50 percent compared to the previous TRN and CO-QBN for different secret key rates, respectively.

As shown in Figure 11, we only analyzed the deployment cost of the HQNC in the FS, with CO-QBN for comparison. From Figure 10 and Figure 11, the total cost of the FS is significantly lower than the total cost value of the CS. The main reason for this is that the definition of each cost value is reduced in the FS from Table 2. Furthermore, the future network cost issue is well predicted.

## 5. Conclusions

We proposed an HTUR QKD network architecture with QKP integrating the MDI-QKD protocol. Moreover, a new HQCN heuristic algorithm was designed to implement the cost optimization in the QKD network. Two scenarios (i.e., CS and FS) were utilized in the simulation to analyze the total cost of QKD requests. The simulation results demonstrated that, in the CS, the cost savings of the HQNC were more than 90 percent and 50 percent compared to the previous TRN and CO-QBN, which showed the effectiveness of the proposed HQNC heuristic algorithm.

## Figures and Tables

**Table 1:** Current actual QKD networks.

Network	Node Number	Relay
DARPA quantum network [16]	10	Trusted relay
		Optical switch
SECOQC quantum network [17]	6	Trusted relay
Tokyo quantum network [18] network	6	Trusted relay
Space-to-ground quantum network [6]	32	Trusted relay

**Table 2:** Abbreviations and definitions.

Abbreviations	Definitions
QKD	Quantum Key Distribution
MDI	Measurement-Device-Independent
QKP	Quantum Key Pool
SDN	Software-Defined Network
HQNC	Hybrid QKD Network Cost
HTUR	Hybrid Trusted and Untrusted Relay
CO-QBN	Cost-Optimized QKD Backbone Networking
PTR	Purely Trusted Relay
API	Application Programming Interface
QTs	Quantum Transmitters
QRs	Quantum Receivers
KM	Key Management
KS	Key Service
OSs	Optical Switches
EDFA	Erbium-Doped Fiber Amplifiers
MOD	Multiplex/Demultiplex
CS	Current Scenario
FS	Future Scenario
PCh	Public Channel
DCh	Data Channel
QCh	Quantum Channel
NSFNET	National Science Foundation Network
USNET	United States Network

**Table 3:** Symbols and definitions.

Symbols	Definitions
G(N,L)	Optical/QKD network topology
*N*	Set of optical/QKD nodes
*L*	Set of QKD links
*m*	The distance between QKD Node 1/trusted relay and untrusted relay
$r(S_{r},d_{r},P_{r})$	A QKD request between two arbitrary distant QKD nodes
*R*	Total QKD requests in QKD backbone network
$P_{r}$	The number of parallel QKD links of r
$E_{r}$	Secret key rate of r
*D*	The distance of a pair of MDI-QTs
$E_{D}$	The secret key rate at distance D
$L_{r}$	Set of fiber links on the path of r
$L_{sd}$	Set of fiber links between QBNs $s_{r}$ and $d_{r}$
γ	The wavelength of QKD link
ω	The wavelength of KM link
$\left(\alpha ,\beta \right)$	Index of QBN nodes in QKD network topology $G\left(N,L\right)$. $\left(\alpha ,\beta \right)\epsilon G\left(N,L\right)$
$W_{Q}$	Set of wavelength channels planned as QChs
$W_{KM}$	Set of wavelength channels planned as KM
$M_{QT}^{r}$	Required number of MDI-QTs for r
$M_{QR}^{r}$	Required number of MDI-QRs for r
$M_{KS}^{r}$	Required number of KSs for r
$M_{MOD}^{r}$	Required number of pairs of Multiplexes/Demultiplexes (MODs) for r
$\delta_{QT}^{r}$	Cost of an MDI-QTs for r
$\delta_{QR}^{r}$	Cost of an MDI-QRs for r
$\delta_{KS}^{r}$	Cost of a KS for r
$\delta_{MOD}^{r}$	Cost of a pair of MODs for r
$\delta_{L}^{r}$	Cost per kilometer of a wavelength channel on a fiber link
$C_{QT}^{R}$	Cost of MDI-QTs for all QKD link requests R
$C_{QR}^{R}$	Cost of MDI-QRs for all QKD link requests R
$C_{KS}^{R}$	Cost of KS for all QKD link requests R
$C_{L}^{R}$	Cost of QKD and KM link for all QKD link requests R
$C_{MOD}^{R}$	Cost of pairs of MODs for all QKD link requests R
$C_{R}$	Total cost of deployment QKD backbone network
$B_{\left(\alpha ,\beta \right),\gamma }^{r}$	Boolean variable that equals 1 if wavelength γ on link (α,β) is assigned to the QKD link of r, and 0 otherwise
$K_{\left(\alpha ,\beta \right),\gamma }^{r}$	Boolean variable that equals 1 if wavelength γ on link (α,β) is assigned to the KM link of r, and 0 otherwise

**Table 4:** Cost values used for the performance evaluation based on QKD networks.

Different Situations	|R|	Scenario	$\delta_{\mathrm{QT}}^{r}$ ($)	$\delta_{\mathrm{QR}}^{r}$ ($)	$\delta_{L}^{r}$ ($)	$\delta_{\mathrm{KS}}^{r}$ ($)	$\delta_{\mathrm{MOD}}^{r}$ ($)
HQNC	|R| > 0	CS	6600	15,000	135	5000	200
		FS	3000	8000	60	2500	100
PTR	|R|> 0	CS	6600	15,000	135	5000	200

## Data Availability

Not applicable.
